# Ethics in academic publishing: a timely reminder

**DOI:** 10.5195/jmla.2017.122

**Published:** 2017-07-01

**Authors:** Frank Houghton

Most, if not all, academic librarians are by now familiar with the term “predatory publishing.” Beall in 2012 defined predatory publishers as “those that unprofessionally exploit the gold open-access model for their own profit,” often taking advantage of junior faculty and graduate students [1]. This problem has grown at an almost exponential rate. Whereas Beall identified 23 suspected predatory publishers in 2011 [1], he identified 1,150 suspect publishers [2] and 1,254 suspect standalone journals [3] as of 2016. As the International Committee of Medical Journal Editors notes, public trust in academic research and the publishing industry is in jeopardy [4].

On an almost daily basis, many academics now receive invitations to publish in journals that Beall correctly identifies as suspect and very probably corrupt. Beall’s open access and alphabetized list makes it extraordinarily easy to investigate and evaluate journals involved in such practices [2, 3]. Of course, for many of us, the initial communication itself has a number of warnings embedded in it that raise red flags. These can include abnormally short publication timelines that cannot possibly reflect the reality of the peer-review and publishing process [5]. Another red flag is a journal’s effusive language toward a potential author, which is in stark contrast to the standard responses that most faculty authors receive from more established and reputable journals.

Another rather obvious warning sign is often the clumsy, poor, or just plain incorrect English grammar that is used in such communications. The following are two examples that the author recently received:

“Being impressed by your quality work, we are contacting you to know if you can associate with us by submitting your upcoming research.”

“Our reputed journal, aspire to deliver high quality research work in the field of health care as Open Access to the globe.”

Based on reflection on the issues of predatory publishing and mindful of current events globally and nationally, it is in relation to this final point that I felt an ethical obligation to write this cautionary piece. Surely there is something wrong when poor English grammar in an email evokes such immediate suspicion.

Before continuing, it is important to note that in 2016 Dictionary.com announced that its word of the year was “xenophobia”:

At Dictionary.com we aim to pick a Word of the Year that embodies a major theme resonating deeply in the cultural consciousness over the prior 12 months. This year, some of the most prominent news stories have centered around fear of the “other.” Fear is an adaptive part of human evolutionary history and often influences behaviors and perceptions on a subconscious level. However, this particular year saw fear rise to the surface of cultural discourse. Because our users’ interest in this overarching theme emerges so starkly for one specific word in our trending lookup data, xenophobia is Dictionary.com’s 2016 Word of the Year. [6]

It is important, therefore, to ensure that concerns over predatory publishing, which may be evoked by something as incidental as poor English, do not develop into a more widespread emotion-heated xenophobia. As White recently noted in response to populist election outcomes globally: “Facts are irrelevant. Emotion and prejudice rule” [7].

Beall’s pioneering work in developing awareness of predatory publishing and in continuing to devote time and energy to this subject is to be admired. However, it is important that whole populations are not indiscriminately written off. Beall’s Scholarly Open Access website contains the following statements in a blog post, titled “Hyderabad, India — City of Corruption,” which are perhaps a cause for concern:

Hyderabad, India is one of the most corrupt cities on earth, I think. It is home to countless predatory open-access publishers and conference organizers, and new, open-access publishing companies and brands are being created there every day. All institutions of higher education, all funders, governments, and researchers should be especially wary of any business based in Hyderabad.

The tacit rule of thumb of Hyderabad-based businesses is: Use the internet to generate revenue any way you can.

There are numerous internet-based businesses in this over-crowded city, many located in a special enterprise zone called HITEC City, which some refer to as “Cyberabad.” [8]

Evidence of this potentially prejudiced appraisal of foreign publications can ironically now be seen in more recent strategies adopted by predatory publishers, including using a “fake” US mailing address and assuming a title falsely indicating that it is a US journal (“*American Journal of So-and-So*”).

However, we must, of course, acknowledge that high-quality non-English-language health and medical journal publications do exist and make a valuable addition to the health sciences. Our ethical duty is to keep an open mind and resist succumbing to ethnocentric prejudice and racism. It is important to remember that it is not only in industrializing nations that publishers have been noted to engage in dubious practices. Even high-status established and reputable academic publishers in the West have been found to be in breach of expected academic norms. Perhaps one of the best examples was the decision by Elsevier Australia to publish six “academic journals” sponsored by the pharmaceutical company Merck (e.g., *Australasian Journal of Cardiology*) [9]. As Ben Goldacre of *The Guardian* notes, “The relationship between big pharma and publishers is perilous” [10], clearly outlining the nature of these publications:

Elsevier Australia went the whole hog, giving Merck an entire publication which resembled an academic journal, although in fact it only contained reprinted articles, or summaries, of other articles. In issue 2, for example, nine of the 29 articles concerned Vioxx, and a dozen of the remainder were about another Merck drug, Fosamax. All of these articles presented positive conclusions. Some were bizarre: such as a review article containing just two references. [10]

Elsevier has condemned this practice [9], but the damage to the credibility of the academic publishing industry remains. Further evidence of sustained assaults by Big Pharma on the world of academic publishing are clearly outlined in Goldacre’s subsequent work, *Bad Pharma: How Drug Companies Mislead Doctors and Harm Patients* [11].

Predatory publishing undoubtedly represents a clear and present danger to the integrity of academic publishing. To date, little has been done to curb the excesses in this field, although some recent action by the US government has been noted [12]. It is important, however, that concerns over predatory publishing do not spiral or morph into an insular, xenophobic rhetoric that smacks of racism. As noted above, even elite publishers such as Elsevier have shown themselves to be swayed by financial returns.
